# Influence of Laser Cutting Parameters on the Microhardness, Roughness, and Microstructure of AISI 304, S355J2, and AlMg_3_ Alloys

**DOI:** 10.3390/ma19020240

**Published:** 2026-01-07

**Authors:** Jaroslaw Selech, Grzegorz Burzynski, Dessie Tibebe, Dariusz Ulbrich, Piotr Banas

**Affiliations:** 1 Institute of Machines and Motor Vehicles, Poznan University of Technology, 60-965 Poznan, Poland; jaroslaw.selech@put.poznan.pl; 2 WKS Spawalnik T. G. Burzyńscy, 64-800 Chodzież, Poland; 3 College of Natural and Computational Science, University of Gondar, Gondar P.O. Box 196, Ethiopia

**Keywords:** cutting, microhardness, S355J2 steel, AlMg_3_, AISI 304 steel, surface roughness

## Abstract

**Highlights:**

**What are the main findings?**

- Optimal surface quality in laser beam cutting requires material-specific optimization of gas pressure.
- Laser beam cutting increases microhardness in the immediate cutting zone across all materials.
- AISI 304 steel shows the highest heat resistance, with no microstructural changes or melt zone observed.

**What are the implications of the main findings?**

- Gas pressure must be tailored to each material to achieve superior surface finish in industrial applications.
- Enhanced microhardness improves wear resistance but may affect ductility in the cut edge.
- AISI 304’s superior thermal stability makes it ideal for high-precision cutting processes requiring minimal distortion.

**Abstract:**

This study provides a comparative and material-specific assessment of how laser cutting parameters affect the surface integrity of three commonly used engineering alloys, thereby extending the current knowledge beyond single-material analyses. The main objective was to quantify and relate changes in surface roughness, microhardness, and microstructure to variations in laser cutting conditions for S355J2 steel, AISI 304 steel, and AlMg_3_ aluminum alloy. Variable cutting parameters were applied, including cutting speed, assist gas type and pressure, as well as laser beam power, and their combined effect on the thickness of the remelted and heat-affected zones was evaluated. The results show clear material-dependent trends: S355J2 steel exhibited the lowest surface roughness but the most pronounced surface hardening, with maximum microhardness values reaching approximately 700 HV 0.1 in a relatively narrow heat-affected zone, whereas AISI 304 showed a distinct edge-hardening effect with more moderate roughness. In contrast, the AlMg_3_ alloy developed a clearly visible remelted layer and a refined, fine-grained microstructure, accompanied by much lower hardness levels but a more diffuse heat-affected zone. These findings provide original, comparative guidelines for selecting laser cutting parameters tailored to specific materials, enabling the optimization of edge quality and surface properties in industrial applications.

## 1. Introduction

With the rapid development of industry, machines are facing ever-increasing demands. As a result, there is a constant drive to improve work quality, efficiency, and reliability, which in turn compels manufacturers to adopt innovative design solutions and production technologies [1]. There are methods that are used to cut elements of the required shapes from steel or aluminum sheets. These methods include cutting with water [2], acetylene–oxygen torches [3,4], punching methods [5], and laser beams [6]. Each method employs different technology, resulting in machined surfaces with distinct properties, including roughness, hardness, heat-affected zone width, and metallographic structure changes [7].

Laser cutting of details is a machining technology characterized by thermal separation of material through sublimation, melting, and burning [8]. The development of laser cutting technology is built upon a rich history of experimental and theoretical research aimed at understanding the thermal processes involved in processing a wide range of materials (from metals like titanium and stainless steel to polymers and ceramics). A key element in the evolution of this method has been the precise optimization of process parameters, such as beam power, scanning speed, and gas pressure, which has allowed for the minimization of technical defects like surface roughness and perpendicularity deviations [9]. A characteristic feature of laser cutting is the localized introduction of energy by a high-energy cutting beam. At higher cutting speeds, laser cutting offers greater precision and is more cost-competitive than methods such as extrusion or casting [10]. Laser cutting has a significant advantage over conventional methods due to the lack of a cutting tool. In addition to its high speed, laser cutting offers other significant advantages, including high processing flexibility and non-contact technology, which ensure precision and repeatability—particularly in large-scale production [11]. Laser cutting enables very high shape repeatability due to laser head positioning accuracy below 0.1 mm and repeatability under 0.05 mm [12]. A laser beam, light amplification by stimulated emission of radiation (LASER), is a quantum generator producing monochromatic, coherent, and polarized light beams [13]. The mechanism of light generation is based on the phenomenon of stimulated emission of radiation in a medium after inversion [14]. Lasers based on carbon dioxide (gas lasers) and crystals (solid-state lasers) are mainly used for cutting [15,16]. In the first stage, the electrons of the molecules are excited to a higher energy level (pumping), after which they rapidly return to their ground state, triggering a high-energy electromagnetic pulse. Repeated pumping and triggering result in the formation of a laser beam [17,18]. The focusing system concentrates the relatively high energy of the laser beam into a small area, generating high temperatures along the cutting path.

Laser beam cutting systems can also be classified by cutting mode. There are two main types depending on laser beam delivery: continuous wave and pulsed [19]. In pulsed cutting, the laser is turned on and off in short intervals. This type of process has advantages because, on average, less heat is generated in the material. Therefore, small holes and complex shapes can be cut with better quality, although the cutting and piercing process is interrupted during breaks between successive pulses, which reduces the cutting speed.

The laser cutting process can be carried out by melting, burning, or sublimation. In melting-mode laser cutting, the material is heated above its melting point by the laser beam and then expelled from the kerf by a stream of inert gas, usually nitrogen [20]. Laser cutting by burning differs from melting because oxygen is used as the assist gas. The interaction of oxygen with the heated metal triggers an exothermic reaction that further increases the material temperature. As a result, cutting speeds for structural steel can be several times higher than in melting mode, particularly for sheets around 6 mm thick [21]. Sublimation cutting is a method in which the material evaporates spontaneously under a high-intensity focused laser beam and/or is ejected by the high pressure of the resulting vapor and assist gas. Sublimation cutting is mainly used for processing wood, plastics, and non-metallic materials [22].

The research presented addresses key aspects of laser cutting technology for various materials. The primary objective was to evaluate basic material properties such as surface roughness, structural changes, and microhardness in response to varying laser cutting parameters such as beam power, gas pressure, and gas type, aiming to identify optimal conditions for maximum hardness and minimal roughness. A literature review revealed a research gap regarding the effects of these parameters on microhardness and surface roughness profiles for S355J2 steel, AISI 304 steel, and AlMg_3_ aluminum alloy. Although laser cutting typically yields clean surfaces requiring no further finishing, suboptimal parameters often lead to material losses, underscoring the need for process optimization with economic considerations. These findings not only advance understanding of laser cutting parameters and their influence on material properties but also provide practical guidance for designing similar processes in construction materials with comparable compositions and characteristics.

## 2. Materials and Methods

This study used three different materials commonly used in the production of machine and equipment components:

1. S355J2 steel—manganese, non-alloy structural steel for general use with increased strength.
2. AISI 304 steel—alloy stainless steel, used, among others, in the machine industry, construction and architecture (e.g., bridge elements, handrails), the automotive industry (exhaust systems, vehicle frames), the food industry (milk, beer, and wine tanks), and the pharmaceutical and cosmetics industries.
3. Aluminum alloy AlMg_3_—an aluminum alloy with magnesium content ranging from 2.6 to 3.6%, which makes it resistant to corrosion; therefore, it is used in the production of structural elements, sheet metal, pressure tanks, as well as in the food and machine industries.

**Figure 1:**
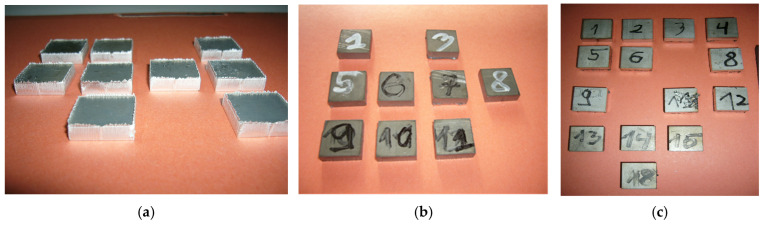
Chosen samples cut from metal sheet and used during the research: (**a**) AlMg_3_, (**b**) S355J2 steel, (**c**) AISI 304 steel.

The chemical composition of the tested materials is presented in Table 1, with selected samples after the cutting process shown in Figure 1.

All tested materials were procured from a commercial wholesaler in the form of sheet stock. Specifically, S355J2 steel sheets were hot-rolled, AISI 304 sheets featured a hot-rolled finish, and AlMg_3_ alloy sheets were provided in the hot-rolled state, with no subsequent heat treatments or surface alterations applied.

**Figure 2:**
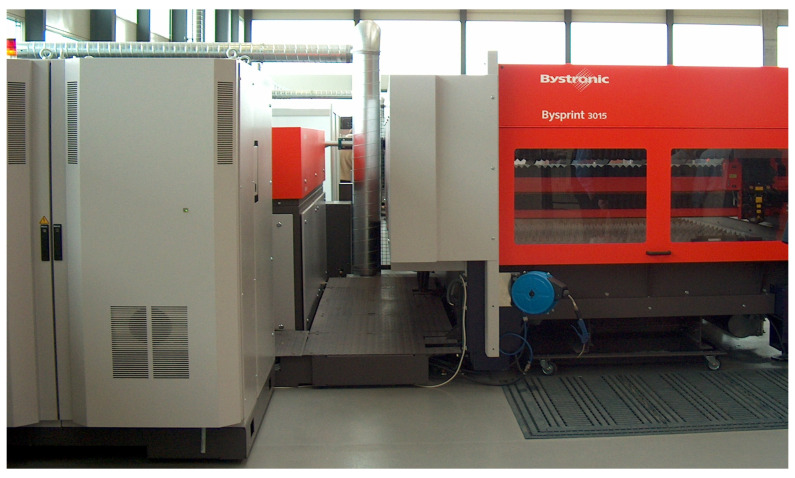
Laser cutting device used for samples cutting process.

Four series of samples were cut for each material, with each series containing 12 samples measuring 20 mm × 20 mm × 5 mm, taking into account changes in the cutting process parameters. A detailed list of samples and their cutting parameters is provided in Table 2. These parameters were selected based on data provided by the manufacturer from the laser cutting machine manufacturer and the authors’ own experience, with cutting performed on a Bystronic BYSPRINT machine (Hoffman Estates, IL, USA), as shown in Figure 2.

The metallographic structure of samples for three types of materials (AlMg_3_, AISI 304, S355J2) was examined under a light microscope, and metallographic examinations were conducted for selected samples. Samples of AISI 304 steel were cut by laser beam, then mounted in epoxy resin. Grinding was performed sequentially from 180 to 1200 grit SiC papers to achieve a planar surface. Polishing used diamond suspensions (9–0.25 µm) followed by alumina to reach a < 1 µm R_a_ finish. Etching with 10% Nital for 10–20 s revealed the austenitic microstructure, with samples examined under an optical microscope. A very similar procedure was applied for S355J2 steel, with the only difference being the use of a lower concentration 2% Nital reagent. For the last material, AlMg_3_, a 0.5% HF solution was applied for 10–20 s. Then, any changes in the microstructure resulting from the laser beam and the heat it generated were verified.

**Figure 3:**
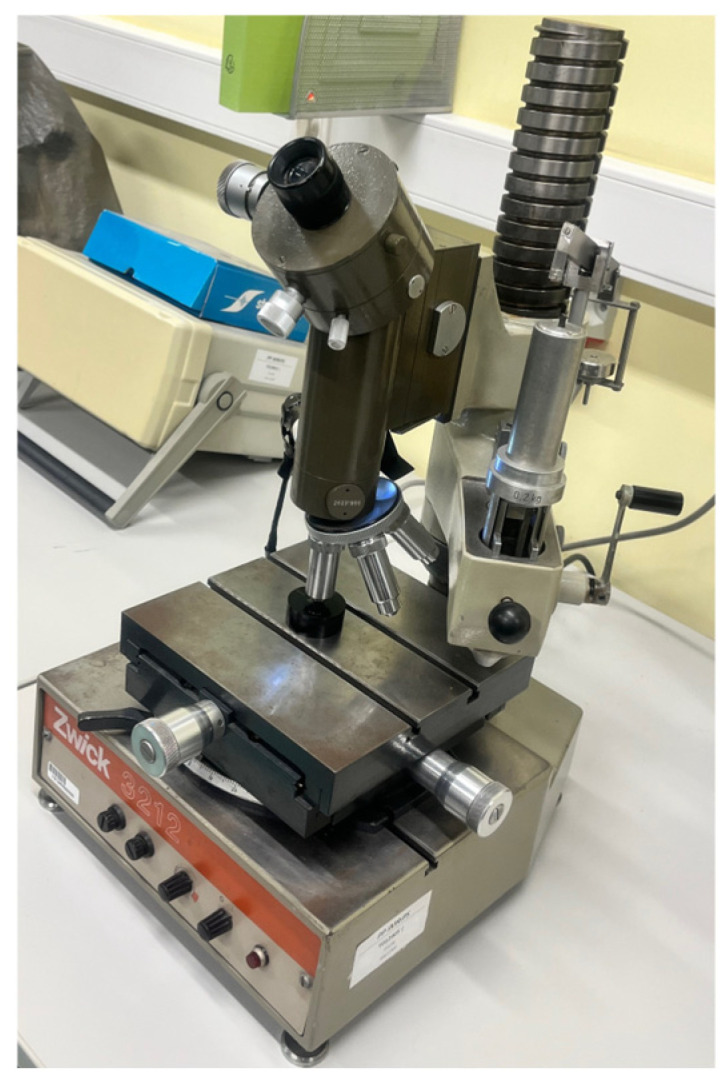
Device used for microhardness measurement.

Microhardness measurements were performed using the Vickers method (HV 0.1) on a classic optical hardness tester Zwick 3212 (Zwick, Ulm, Germany), featuring a diamond pyramid indenter (136° angle) and microscopic measurement of indentation diagonals (Figure 3). These measurements followed ISO 6507-1 standards [24], involving a 100 gf (0.981 N) load applied for 10–15 s, with at least 5 indents per position for repeatability and diagonal averaging via filar eyepiece optics. The HV 0.1 was selected for tested materials due to their moderate hardness range, enabling precise near-edge profiling (0.05–3 mm) without excessive indentation size (>20 µm diagonal needed), minimizing substrate effects in thin HAZ zones, and ensuring compliance with microhardness norms for metallic alloys. This allowed for the influence of individual cutting process parameters on the change in hardness in the edge areas to be determined. This is particularly important for small components whose properties can be significantly altered by the manufacturing process. The same procedure was applied to all samples, for which microhardness was measured at various distances from the cut edge.

**Figure 4:**
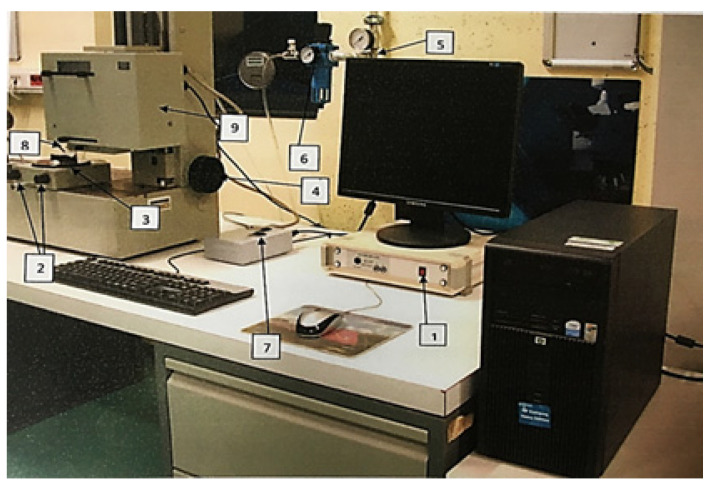
Device used for surface profile measurement; 1—Roform Amplifier panel switch, 2—knobs for leveling the measuring table and setting the measurement point, 3—measuring table, 4—knob for lowering and raising the sensor head, 5—compressed air shut-off valve, 6—pressure reducer with compressed air dryer, 7—key for controlling fast movement of the measuring sensor, 8—measuring sensor with needle, 9—measuring head; developed based on.

Surface roughness is a characteristic of the surface of a solid body, meaning recognizable optical or mechanically detectable surface irregularities that do not result from its shape, but are at least one order of magnitude smaller. The values of two basic parameters of the surface roughness profile, R_a_ (average surface roughness for the length of the measurement) and R_z_ (an average of the five largest total deviations recorded for length), were verified on the cut surface. The tests were performed at several points on the cut surface using a ME-10 profilometer (Carl Zeiss, Oberkochen, Germany), and the final result was taken as the average of 5 measurements. The stand used for roughness profile measurement is presented in Figure 4.

## 3. Results and Discussion

### 3.1. Microstructure

**Figure 5:**
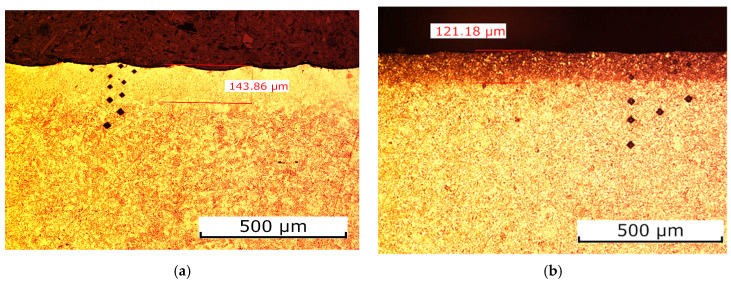

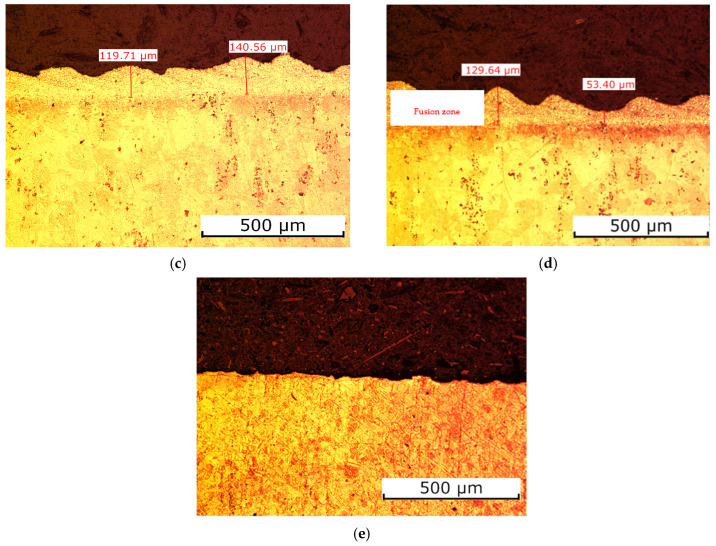
Microstructure of tested materials: (**a**) S355J2 steel—sample 5, (**b**) S355J2 steel—sample 8, (**c**) AlMg_3_ alloy—sample 9, (**d**) AlMg_3_ alloy—sample 10, (**e**) AISI 304 steel.

The microstructure of the materials was verified, as shown in Figure 5 (selected examples from the entire sample series). The core structure of S355J2 steel is fine-grained ferritic–pearlitic. Figure 5a (sample 1) reveals clear metallographic changes due to laser beam heating. Analysis shows that the core structure of sample 5 shifted from ferritic–pearlitic to ferritic–austenitic with minor austenite content, with no distinct melt zone observed. The microstructure change extended 143 µm from the laser-treated edge into the material. Figure 5b also displays the ferritic–pearlitic core structure; however, comparing sample 5 with sample 8 reveals edge changes from laser treatment. The key difference is the affected distance: 121 µm for sample 8. Reducing laser power from 1800 W (sample 5) to 1600 W and increasing gas pressure from 0.5 bar to 0.8 bar altered the edge structure from ferritic–pearlitic to one with higher pearlite content (dark areas of cementite and ferrite), with no clear melt zone visible, as in sample 5.

The AlMg_3_ aluminum alloy is characterized by a coarse-grained structure. Figure 5c,d show the melt zone—there was clear material flow. Sample 9 (Figure 5c) was cut with a beam power of 4000 W and a pressure of 16 bar (the maximum possible pressure at which the cutting process took place). It can be observed that the melt zone distance in this sample is more stable than in sample 10 (Figure 5d), measuring approximately 14 µm. However, structural changes extend about 150 µm from the cutting edge into the material. The cutting process and parameters produced a fine-grained structure. Sample 10, cut at 4000 W beam power and 4 bar pressure (the lowest pressure tested), exhibits a coarse-grained structure similar to series 9. In this case, the appearance of the melt zone occurred at a distance of approx. 9 µm from the edge treated with the laser beam. The average distance at which structural changes occurred was approx. 130 µm. The surface view of this sample also shows increased roughness caused by the use of low pressure during the cutting process.

Figure 5e shows a sample made of AISI 304 steel. The core of this steel is characterized by a predominantly austenitic structure in a hot-treated state. As can be seen from the figure, AISI 304 steel is characterized by high heat resistance due to the lack of noticeable changes in the metallographic structure. No remelting zone was observed, and no changes can be seen at the edge of the laser-treated area. This is due to the alloying additives that make up the structure of this steel, whose task is to prevent the steel from oxidizing under the influence of temperature.

For the three materials considered above, typical literature and datasheet values can be adopted for the purposes of thermal analysis [25,26]:

- For AISI 304, the melting temperature is approximately 1400–1450 °C, with a thermal conductivity of about 16 W/m × K around 100 °C (increasing to roughly 20–21 W/m × K at elevated temperatures);
- For S355J2, the melting temperature is typical of low-alloy carbon steels, around 1460–1520 °C (often approximated as 1490 °C), with a thermal conductivity in the range of 45–55 W/m × K;
- For the AlMg_3_ alloy, the melting temperature is about 600–650 °C (commonly taken as 630 °C), while its thermal conductivity is on the order of 120–150 W/m × K, typically 130 W/m × K.

This information is fully sufficient to justify differences in the temperature field and heat-affected zone depth in the laser cutting analysis. Differences in melting temperature and thermal conductivity directly explain the observed shape and thickness of the remelted zones and heat-affected zones in the presented microstructures. For S355J2 steel, the melting temperature is high, and the thermal conductivity is moderate, which means that very high local temperatures are required to achieve melting, while heat removal into the core is relatively slow. As a result, a relatively narrow but clearly defined remelted zone (on the order of 140 µm) and a not-very-deep heat-affected zone are formed, which is exactly what is seen in the S355J2 steel image, where the boundary between the remelted layer and the base material is distinct. In the case of the AlMg_3_ alloy, the situation is opposite in terms of material parameters: it has a much lower melting temperature and a much higher thermal conductivity. The lower melting temperature promotes easier and potentially deeper remelting, but at the same time, the very high thermal conductivity causes rapid dissipation of energy into the bulk of the material and laterally. In practice, this means that with appropriately selected laser parameters, a similar thickness of the remelted zone can be obtained (approximately 120–140 µm), but the accompanying heat-affected zone is more “diffuse”—the temperature and microstructure gradient extends over a larger distance.

### 3.2. Surface Roughness Profile

**Figure 6:**
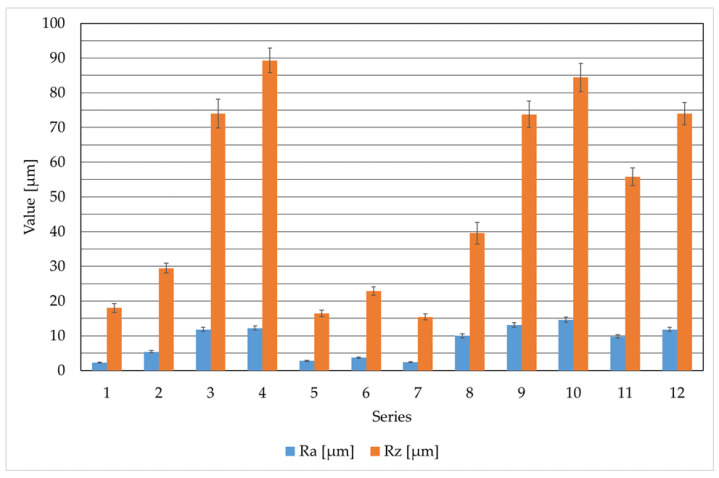
Average results of surface roughness profile parameters for all 12 measurement series for different materials.

Surface roughness profile tests were performed on the samples to verify the impact of selected cutting process parameters on the condition of the surface layer. Post-cutting measurement results were compared to R_a_ and R_z_ values of the materials in their initial state, with average surface roughness profile values for all sample series summarized in Figure 6. For AISI 304 in its initial state, the baseline parameters were R_a_ = 1.1 µm and R_z_ = 7.4 µm. These represented the lowest average surface roughness values among all tested samples. No variation in cutting parameters, gas pressure, or beam power produced a smoother surface. The highest roughness values occurred in series 4 samples, cut with oxygen at maximum pressure. Series 1 samples (using manufacturer-recommended parameters) exhibited surface roughness closest to the raw material. The samples from series 2 were cut at four times lower pressure than the maximum possible pressure, but their R_a_ and R_z_ roughness coefficients increased only about twice compared to series 1. This resulted in significantly lower gas consumption with a slight increase in surface roughness. This pressure value represented the minimum threshold below which material cutting did not occur. Oxygen-assisted cutting produced the highest average R_a_ and R_z_ values, which are undesirable for cutting equipment users. These values and the surface condition were characteristic of the plasma cutting process [27].

The S355J2 steel, whose surface was not subjected to cutting, was characterized by average roughness profile values of R_a_ = 3.9 µm and R_z_ = 19.2 µm. The S355J2 structural steel was cut only in an oxygen shield. In practice, other gases are not used for cutting this type of steel [28,29]. Based on the analysis of the obtained results, the surface roughness profile parameters for series 8 samples are nearly three times higher than those for series 5 and 7, and almost twice as high as for series 6. The analysis of the results of laser beam cutting of S355J2 steel (at a constant cutting speed and cutting gas (oxygen)) clearly indicates the key influence of gas pressure on surface roughness (R_a_ and R_z_). The best surface quality (lowest roughness: R_a_ = 2.4 µm, R_z_ = 15.4 µm) was achieved for samples from series 7. Increasing gas pressure at constant power significantly deteriorated surface quality, with series 8 samples representing the extreme case of the worst roughness. This indicates that excessive pressure—likely causing uncontrolled molten material ejection—negatively impacts cut edge smoothness, as evidenced by elevated R_a_ and R_z_ values.

Samples made of AlMg_3_ material were cut in a nitrogen shield, as oxygen is not used for this material. The four test series of samples were cut at constant pressure with varying laser beam power and at constant laser beam power with varying pressure. The material in its delivery state was characterized by the following surface roughness values: R_a_ = 0.5 µm and R_z_ = 5.2 µm. These are the lowest surface roughness values of all the results obtained. A detailed analysis of the cutting results for this material at a constant speed using nitrogen showed that the best surface quality (lowest roughness: R_a_ = 9.8 µm, R_z_ = 55.8 µm) was achieved for samples from series 11 with the parameters. The results indicate a complex optimization, where increasing the laser power from 3600 W to 4400 W (at constant gas pressure) deteriorated surface quality (R_a_ increased to 11.8 µm). This may be due to excessive energy input during cutting. At the same time, too low nitrogen pressure (4 bar in series 10) proved to be the worst set of parameters (R_a_ = 14.6 µm), confirming that in laser beam cutting, nitrogen must have a pressure of 14 bar and above to effectively remove molten metal at the cutting site.

Variations in R_a_ and R_z_ roughness result from the combined influence of the laser beam energy balance, melting and rapid solidification phenomena, and the effectiveness of molten metal removal by the assist gas [30]. Higher R_a_ and R_z_ values are interpreted as the effect of a more intense oxidation reaction, a deeper molten pool, an unstable melt front, and turbulent ejection of liquid material, which leads to deeper grooves and increased waviness on the cut surface. This interpretation has been linked to published models of laser cutting of steels and aluminum alloys, which emphasize the role of gas pressure, oxidation, liquid metal flow, and solidification rate in shaping roughness parameters [10,31]. The authors’ work and the study by Librera et al. [32] show that laser cutting conditions significantly affect surface roughness and cut-edge quality, and that a reliable assessment of this roughness requires carefully selected measurement parameters and procedures that account for the strong dependence of R_a_/R_z_ on measurement position and cut-off settings.

### 3.3. Microhardness

**Figure 7:**
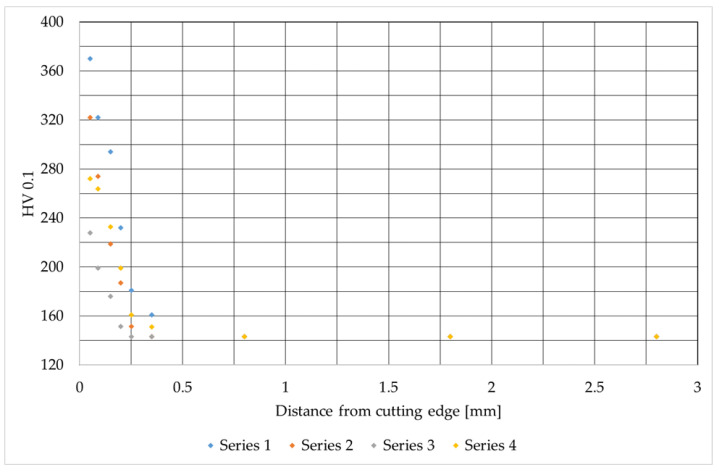
Microhardness values as a function of distance from the cutting edge for AISI 304 steel.

**Figure 8:**
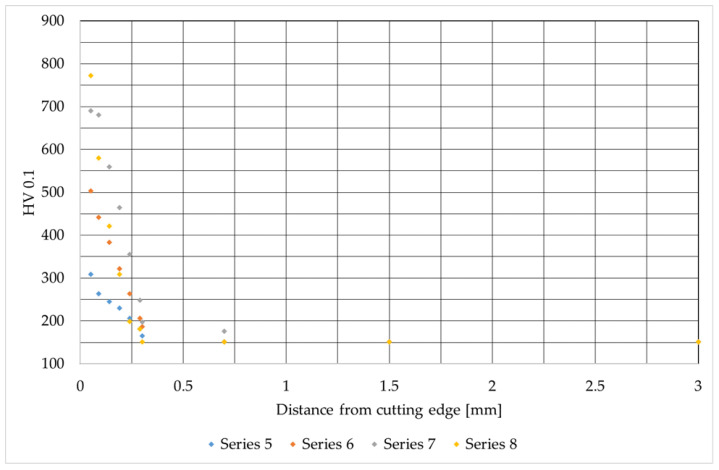
Microhardness values as a function of distance from the cutting edge for S355J2 steel.

**Figure 9:**
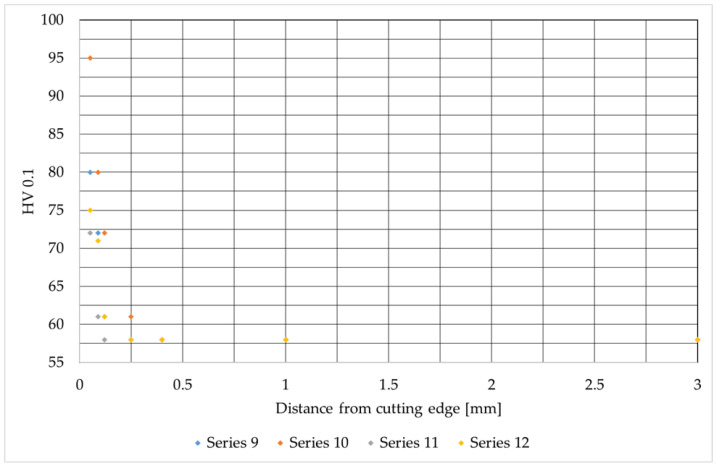
Microhardness values as a function of distance from the cutting edge for AlMg_3_ alloy.

This stage of the research involved assessing the microhardness across the sample surfaces, measured from the cut edge into the material. The results of these tests are presented in Figure 7, Figure 8 and Figure 9. They are consistent with the results available in the literature [30,33].

Figure 4 shows the distribution of microhardness (HV 0.1) as a function of distance from the cutting edge for AISI 304 material, revealing clear edge hardening resulting from the machining process. The base material hardness stabilizes at approximately 0.8 mm from the edge, averaging 145 HV 0.1. Directly at the cutting edge, hardness increases rapidly, reaching a maximum of approximately 370 HV 0.1 in samples from series 1. This represents an increase of more than 155% from the core microhardness, indicating the most intense hardening effect. The samples from series 4 achieve the lowest hardness at the cutting edge, equal to approximately 230 HV 0.1. Despite differences in hardening intensity, the critical zone of influence (where hardness returns to base level) remains similar across all series at approximately 0.4 mm from the edge. The analysis of the graph shows a clear difference between samples cut with nitrogen and samples cut with oxygen. Samples from series 1, 2, and 3 were cut with nitrogen, and an increased microhardness is clearly visible for these series.

Analysis of the microhardness results for S355J2 steel after oxygen cutting shows that immediately at the cutting edge (up to approx. 0.2 mm), the hardness increases rapidly, reaching values from approx. 300 HV 0.1 (test 5) to over 700 HV 0.1 (test 8), then drops very quickly and stabilizes at around 150 HV 0.1 deeper into the material. This indicates a strong influence of heat treatment and local overheating at the surface, as well as varying effects based on process parameters. The highest hardness values and most extensive heat-affected zone occur in test 8, corresponding to the highest oxygen flow from prior data. This confirms that intense cutting conditions lead to excessive surface hardness and deterioration of the steel’s structural quality.

Samples from series 12 (aluminum alloy) were cut at a maximum beam power of 4400 W and at almost maximum gas pressure of 14 bar (max 16). With these cutting process parameters, the highest hardness of 95 HV 0.1 was achieved. The microhardness of the core only appears at a distance of approx. 0.4 mm from the cut edge. Samples from series 11 achieve the lowest average microhardness results with cutting parameters of 3600 W and a pressure of 14 bar. These parameter settings are recommended by the device manufacturer. Therefore, they should be considered the standard starting settings for cutting aluminum alloys. The maximum microhardness in this case is 72.5 HV 0.1, and values close to the microhardness of the core appear at a distance of approx. 0.12 mm from the cutting edge.

Samples from series 9 and 10 exhibit similar microhardness profiles, differing only in their initial values at the cutting edge. At a distance of approximately 0.09 mm from the edge, these values converge and remain consistent over the further measurement range.

Differences in the depth of the heat-affected zone for S355J2 and AlMg_3_ correlate well with the microhardness profiles and the thermal properties of both materials. In S355J2 steel, due to its high melting temperature and moderate thermal conductivity, a narrow but clearly defined remelted and heat-affected zone (about 0.1–0.4 mm) is formed, accompanied by a sharp increase in hardness at the edge (up to more than 700 HV 0.1) and a steep gradient into the material. In the AlMg_3_ alloy, with a much lower melting temperature and high thermal conductivity, a similar thickness of the remelted layer is obtained, but the energy distribution is more gradual, resulting in lower maximum microhardness values (about 70–95 HV 0.1) and a more diffuse heat-affected zone, where hardness close to that of the core appears already at a distance of 0.09–0.4 mm from the edge.

## 4. Conclusions

Based on multi-stage tests of three different materials subjected to laser beam cutting, the following conclusions can be drawn:

- Laser cutting technology, characterized by high precision, repeatability, and flexibility in parameter selection, introduces significant changes in the surface layer properties of cut materials, primarily in surface roughness, microstructure, and microhardness.
- For oxygen-cut structural steels, excessive assist gas pressure is detrimental, as it drastically worsens surface roughness (S355J2) and causes pronounced hardening in the zone directly adjacent to the cut edge.
- All materials exhibited edge hardening up to a depth of approximately 0.4 mm from the cutting edge, with the highest value of approximately 700 HV 0.1 recorded for S355J2 steel (series 8). In contrast, series 1, i.e., AISI 304 steel, showed the highest relative increase in hardness of approximately 155% compared to the microhardness of the core.
- For S355J2 steel, assist gas pressure proved to be a key factor affecting the surface layer. Excessive pressure (0.8 bar, series 8) drastically worsened roughness, probably due to uncontrolled removal of molten material. The best surface quality was achieved at the lowest pressure of 0.1 bar in series 7.
- For AISI 304 steel, the highest heat resistance was obtained, with no noticeable changes in microstructure and no melt zone. Completely opposite conclusions were noted for the aluminum alloy: a distinct melt zone formed, and the material structure changed to a finer-grained one.

Since laser beam cutting is widely used for various materials, understanding its impact on surface layer properties is crucial. This article and the conclusions presented are part of this thematic area and may serve as a basis for estimating the properties of the surface after the cutting process for those who require specific parameters of surface roughness and microhardness. In the next stages of the research, the authors plan to conduct similar studies for other materials and compare the results obtained with those for other cutting technologies.

## Figures and Tables

**Table 1:** Chemical composition of tested materials [23].

Steel	C	Mn	Si	P	S	Ni	Al	Cr	Mg	Fe
S355J2	0.20	1.50	0.55	0.035	0.035	0.3	0.02	0.3	-	balance
AISI 304	0.08	2.0	0.75	0.045	0.030	9–11	-	18–20	-	balance
AlMg_3_	0.10	0.50	0.40	0.030	0.010	-	balance	-	3.6	-

**Table 2:** Samples and laser cutting parameters.

Material	Series	Gas	Laser Beam Power	Gas Pressure	Cutting Speed
AISI 304	1	Nitrogen	2200 W	16 bar	2200 mm/min
	2	Nitrogen	2200 W	4 bar	2200 mm/min
	3	Nitrogen	1900 W	6 bar	2200 mm/min
	4	Oxygen	2200 W	10 bar	2200 mm/min
S355J2	5	Oxygen	1800 W	0.5 bar	2200 mm/min
	6	Oxygen	1600 W	0.3 bar	2200 mm/min
	7	Oxygen	1600 W	0.1 bar	2200 mm/min
	8	Oxygen	1600 W	0.8 bar	2200 mm/min
AlMg_3_	9	Nitrogen	4000 W	16 bar	1800 mm/min
	10	Nitrogen	4000 W	4 bar	1800 mm/min
	11	Nitrogen	3600 W	14 bar	1800 mm/min
	12	Nitrogen	4400 W	14 bar	1800 mm/min

## Data Availability

The original contributions presented in this study are included in the article. Further inquiries can be directed to the corresponding authors.
